# Geographical and climatic gradients of evergreen versus deciduous broad‐leaved tree species in subtropical China: Implications for the definition of the mixed forest

**DOI:** 10.1002/ece3.2967

**Published:** 2017-04-13

**Authors:** Jielin Ge, Zongqiang Xie

**Affiliations:** ^1^ State Key Laboratory of Vegetation and Environmental ChangeInstitute of BotanyChinese Academy of SciencesXiangshanBeijingChina

**Keywords:** deciduousness, extreme cold temperature, forest delimitation, leaf type, mixed forest, transitional zone

## Abstract

Understanding climatic influences on the proportion of evergreen versus deciduous broad‐leaved tree species in forests is of crucial importance when predicting the impact of climate change on broad‐leaved forests. Here, we quantified the geographical distribution of evergreen versus deciduous broad‐leaved tree species in subtropical China. The Relative Importance Value index (RIV) was used to examine regional patterns in tree species dominance and was related to three key climatic variables: mean annual temperature (MAT), minimum temperature of the coldest month (MinT), and mean annual precipitation (MAP). We found the RIV of evergreen species to decrease with latitude at a lapse rate of 10% per degree between 23.5 and 25°N, 1% per degree at 25–29.1°N, and 15% per degree at 29.1–34°N. The RIV of evergreen species increased with: MinT at a lapse rate of 10% per °C between −4.5 and 2.5°C and 2% per °C at 2.5–10.5°C; MAP at a lapse rate of 10% per 100 mm between 900 and 1,600 mm and 4% per 100 mm between 1,600 and 2,250 mm. All selected climatic variables cumulatively explained 71% of the geographical variation in dominance of evergreen and deciduous broad‐leaved tree species and the climatic variables, ranked in order of decreasing effects were as follows: MinT > MAP > MAT. We further proposed that the latitudinal limit of evergreen and deciduous broad‐leaved mixed forests was 29.1–32°N, corresponding with MAT of 11–18.1°C, MinT of −2.5 to 2.51°C, and MAP of 1,000–1,630 mm. This study is the first quantitative assessment of climatic correlates with the evergreenness and deciduousness of broad‐leaved forests in subtropical China and underscores that extreme cold temperature is the most important climatic determinant of evergreen and deciduous broad‐leaved tree species’ distributions, a finding that confirms earlier qualitative studies. Our findings also offer new insight into the definition and distribution of the mixed forest and an accurate assessment of vulnerability of mixed forests to future climate change.

## 1 Introduction

Evergreen and deciduous broad‐leaved tree species can coexist across a variety of landscapes around the globe and play important roles in forest structure and functions (Kikuzawa, Onoda, Wright, & Reich, 2013; Ouédraogo et al., 2016; Wang, Kent, & Fang, 2007). The relative dominance of different leaf types (evergreen vs. deciduous) is of key importance to the physiognomy of some forests and often provides a main axis for vegetation classification into evergreen forests, deciduous forests, or mixed forests (Franklin et al., 2015; Wu, 1980). Variation in the fraction of evergreenness versus deciduousness has many cascading effects on ecosystem functioning across a variety of forests. For example, shifts in forest characteristics from deciduous to evergreen dominance have been shown to contribute to seasonal changes in energy, water, and carbon balance that have been reported in many tropical and subtropical forests (Bohlman, 2010; Ge, Wang, Xu, & Xie, 2016; Singh & Kushwaha, 2016; Zhou et al., 2014). Therefore, an in‐depth understanding of how geographical patterns in evergreenness and deciduousness are affected by climate will improve our ability to predict how vegetation patterns will respond to future climate change (Ge et al., 2015; Ouédraogo et al., 2016; Xie, Wang, & Silander, 2015).

Patterns in the dominance of evergreen and deciduous tree species, and environmental factors influencing these patterns, have intrigued plant ecologists, biogeographers, and global modelers for decades (Bowman & Prior, 2005; Givnish, 2002; Kikuzawa et al., 2013; Monk, 1966). Evergreen and deciduous species often exhibit contrasting ecological strategies to cope with different climates (Givnish, 2002; van Ommen Kloeke, Douma, Ordoñez, Reich, & Van Bodegom, 2012; Weng, Farrior, Dybzinski, & Pacala, 2016; Wright et al., 2004). For example, long‐lived leaves of evergreen species potentially allow for a longer photosynthetic season than the leaves of deciduous species allow, while deciduous species reduce transpiration and respiration during drought or low temperature and usually possess higher photosynthetic rates per unit leaf area (Aerts, 1995; Givnish, 2002; González‐Zurdo, Escudero, Babiano, García‐Ciudad, & Mediavilla, 2016; Kikuzawa et al., 2013). These pronounced advantages and disadvantages of the respective leaf types are closely coupled with the distribution of evergreen and deciduous tree species in divergent climatic conditions (Condit et al., 2000; Weng et al., 2016; Woodward, Lomas, & Kelly, 2004). For example, Bowman and Prior (2005) established a link between climate and evergreen and deciduous vegetation in seasonally tropical regions of Australia and found that seasonal variability in precipitation may account for the predominantly evergreen woody vegetation of northern Australia. However, other studies have shown a tight relationship between forest deciduousness and/or evergreenness and temperature‐related variables such as mean annual temperature (Kikuzawa & Lechowicz, 2011; Zhang, Luo, Zhu, Daly, & Deng, 2010).

These published studies have greatly advanced our understanding of how climate impacts spatial patterns in the relative dominance of evergreen versus deciduous species in forests. However, the strength and direction of these relationships need further assessment to improve predictions of climate change effects on forest ecosystems. In addition, our understanding of climate influences on the patterns in the dominance of evergreen versus deciduous species within forests remains incomplete in the following aspects (Kikuzawa & Lechowicz, 2011). First, most studies have focused on quantification of leaf life span at the species level, but overlook community‐scale patterns (Kikuzawa et al., 2013; Zhang et al., 2010). Second, although the general dominance patterns of evergreen versus deciduous broad‐leaved tree species along climatic gradients have been qualitatively explained by previous studies (Tang, 2015; Wu, 1980), much more attention has been devoted in tropical regions where water stress is often recognized as a key determinant of these patterns (Bohlman, 2010; Ouédraogo et al., 2016; Singh & Kushwaha, 2016). Few studies have documented the quantitative relationship between climate and evergreen versus deciduous species dominance in subtropical broad‐leaved forests, especially in China. Third, while some evidence suggests that climate influences evergreen versus deciduous dominance in broad‐leaved forests (Suzuki, Ishihara, & Hidaka, 2015; Zhou et al., 2014), the relative contribution of individual climatic variables to the spatial distribution of evergreen and deciduous broad‐leaved tree species and the mechanisms driving these distributions remain largely unclear (Givnish, 2002; van Ommen Kloeke et al., 2012; Ouédraogo et al., 2016).

Evergreen and deciduous broad‐leaved tree species are fundamental components of zonal forests in subtropical China. As latitude increases, these zonal forests gradually transition from evergreen broad‐leaved forests (EBF) to mixed evergreen and deciduous broad‐leaved forests (MEDBF), where evergreen and deciduous species codominate, and finally to deciduous broad‐leaved forests (DBF) (Wu, 1980). Given their high biodiversity and small geographical range in the world, EBF, MEDBF, and DBF forests are among the globe most vulnerable ecosystems to climate change (Ge et al., 2015; Myers, Mittermeier, Mittermeier, Da Fonseca, & Kent, 2000; Seddon, Macias‐Fauria, Long, Benz, & Willis, 2016). Therefore, a better understanding of how climate influences these forests is urgently required to predict the long‐term responses to imminent climate change in subtropical regions (He & Soden, 2017). Such quantitative studies are surprisingly lacking, in spite of well‐established descriptions of these forests at local scales (Ge et al., 2015; Wang et al., 2007).

Furthermore, although distribution patterns of these three forest types along the geographical latitudinal gradient have been described, the boundary conditions separating MEDBF and the adjacent EBF and DBF are not well understood. One of critical hurdles is that no quantitative definition has been explicitly proposed to distinguish MEDBF from EBF and DBF, which may result in contradictions among reports (The Editorial Board of Chinese Forests, 2003). This lack of explicit definitions distinguishing forest type also has led to difficulties in assigning vegetation types to a specific broad‐leaved forest in particular, even though this forest is among those of major interest in ecological research on the effects of global warming (Ge et al., 2015; Song, Kohyama, & Da, 2014).

In this study, we explored the influence of climate on regional patterns in the dominance of evergreen and deciduous species within broad‐leaved forests by compiling data collected specifically for this study with previously collected data from the same study area in subtropical China. Specifically, we aimed to (1) quantify latitudinal distribution of evergreen versus deciduous broad‐leaved tree species, (2) identify the relative contribution of climatic factors to observed patterns, and (3) develop definitions for the three forest types and the climatic thresholds separating them based on the findings for objective 1 and objective 2.

## 2 Methods

### 2.1 Data collection

We assembled a species composition database of subtropical Chinese forests observed in this study and reported in peer‐reviewed papers and monograph publications (see Appendix S1 for more detail of data sources). For analysis, the database was strictly filtered for forest plots located in subtropical regions in China according to the following criteria: (1) All plots were zonal forests and, in order to minimize effects of forest succession, located in natural climax forest stands (zonal vegetation), which we defined as stands which had developed with relatively low disturbance and no evidence of fire, flood, storm, or insect damage or forest management. We also excluded some high‐altitude natural forests at low latitudes. (2) In order to allow for direct cross‐site comparisons and avoid overestimating the relative important value index (RIV) among different plots, only records from published sources that sampled plot sizes ≥0.04 ha during their initial census and that contained RIV estimates for both evergreen broad‐leaved species (EBT) and deciduous broad‐leaved species (DBT) were included in the analyses. The classification of evergreen and deciduous tree species was based on source publications or local flora reference guides. (3) In order to allow determination of climatic data, only sites of known geographical locations were included. (4) All forest plots within Karst regions (edaphic climax) were excluded. Following our filtering criteria, we could maximize the tight link between broad‐leaved forests and climatic variables (Luo et al., 2002; Wu, 1980). After filtration of the database to meet the above criteria, a total of 54 study sites were included in the analyses. The location of analyzed sites ranged from 23.5° to 34.0°N in latitude and from 103.1° to 121.8°E in longitude. See Appendix S2 for additional information.

In our compiled species composition database, we used the Relative Importance Value index (RIV) to quantify the rank of species composition in forests in the ordinary manner. The RIV can be calculated as the average of relative density, relative frequency, and relative basal area (Monk, 1966; Song et al., 2014). We adopted this formula for calculation of RIV because it is widely used and allows easy cross‐site or cross‐study comparisons (Scientific Committee of Chinese Ecosystem Research Network, 2007).

### 2.2 Data processing and analysis

To explain variation in the relative distribution of evergreen versus deciduous broad‐leaved tree species across subtropical China, we selected three explanatory variables that had previously been shown to affect either evergreen versus deciduous prevalence in forests (see Appendix S3 for detailed biological and statistical justifications of these explanatory variables): mean annual temperature (MAT), minimum temperature of the coldest month (MinT), and mean annual precipitation (MAP). MinT is usually used as a surrogate for the annual extreme low temperature (Harrison et al., 2010). Long‐term averages for climatic variables were estimated using the WorldClim Version 1.4 database (Hijmans, Cameron, Parra, Jones, & Jarvis, 2005). Potential evapotranspiration is usually modeled using WorldClim Version 1.4 database (Zomer, Trabucco, Bossio, & Verchot, 2008). As the potential evapotranspiration was highly correlated with the selected climatic variables, it was discarded and not considered in subsequent analyses.

In order to examine latitudinal and climatic patterns for EBF and DBF across the subtropical region, we conducted multiple regression analyses, using RIV of EBT or DBT as response variables, and latitude, MinT, MAT, and MAP and their respective square and cubic functions as candidate explanatory variables. The relationship between the response and explanatory variables was fitted using linear, second‐order polynomial, and third‐order polynomial models. We selected best‐fit models by calculating Akaike's information criterion (AIC) (Aho, Derryberry, & Peterson, 2014) and based on earlier similar studies (Song et al., 2014; Suzuki et al., 2015). Here, we adopted third‐order polynomial models to explore the relationships between the relative distribution of evergreen and deciduous broad‐leaved tree species with latitude and climatic variables. A more detailed explanation and statistical results can be found in Appendix S4.

We also conducted variation partitioning analysis in order to determine the degree to which the relative dominance of evergreen and deciduous broad‐leaved tree species were explained by the above‐mentioned selected climatic variables. The climatic variable importance assessment was based on the best‐fit models using the independent variables mentioned above and forward stepwise model selection. Using the best‐fit model, the variation in RIV of evergreen broad‐leaved tree species was partitioned into individual and interactive effects of the above‐mentioned climatic variables. This variation partitioning analysis followed the statistical guidance provided by Ray‐Mukherjee et al. (2014). More details can be found in Appendix S4. We performed all statistical analyses in R software (R Core Team, 2013).

## 3 Results

### 3.1 Latitudinal patterns

**Figure 1:**
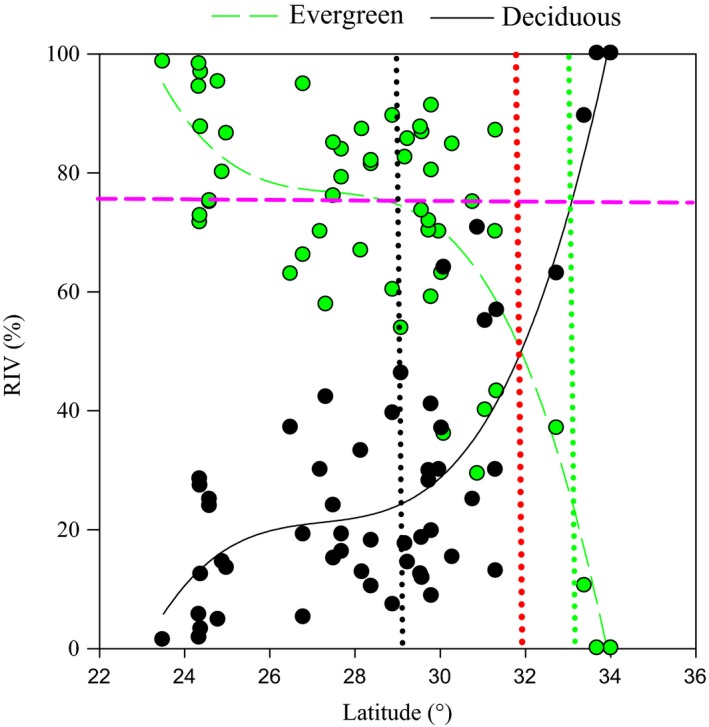
Variation in the relative importance value (RIV) of evergreen and deciduous broad‐leaved tree species with latitude in subtropical China. The interval between the green and black vertical dotted lines is the estimated latitudinal range of the mixed evergreen and deciduous broad‐leaved forest proposed in our study (see text for more detail). The green and black vertical dotted lines indicate the potential upper and lower limits of this mixed forest, respectively. The red vertical dotted line indicates the potential optimum latitudinal location where the RIV of evergreen and deciduous broad‐leaved tree species equals 50%, namely the optimal distribution location for the target mixed forest distribution. The pink horizontal dash line indicates where the RIV of evergreen or deciduous broad‐leaved tree species was equal to 75%

There was a clear south‐to‐north gradient in variation in the dominance of evergreen and deciduous species as indexed by the Relative Importance Value (RIV) (Figure 1). Specifically, we found RIV of the evergreen species to decrease with latitude at a lapse rate of *ca*. 10% per degree between 23.5 and 25°N, *ca*. 1% per degree between 25 and 29.1°N, and *ca*. 15% per degree at 29.1–34°N. The critical location where evergreen species were equal in dominance to deciduous species was at *ca*. 32°N in subtropical China.

### 3.2 Climatic patterns

**Figure 2:**
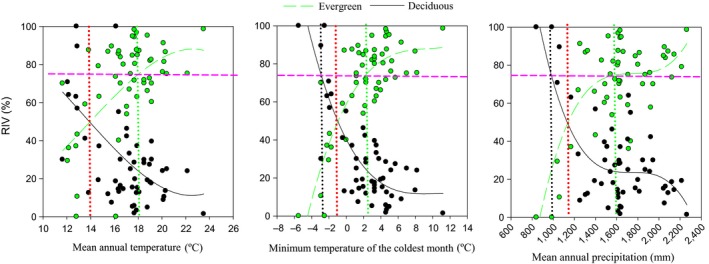
Variation in the relative importance value (RIV) of evergreen and deciduous broad‐leaved tree species with different climatic variables in subtropical China. The intervals between the green and black vertical dotted lines are climatic envelopes of the mixed evergreen and deciduous broad‐leaved forest proposed in our study (see text for more detail). The green and black vertical dotted lines indicate the potential upper and lower limits of the evergreen and deciduous broad‐leaved mixed forest, respectively. The red vertical dotted lines indicate the optimum climatic conditions where the RIV of evergreen and deciduous broad‐leaved tree species equals 50%, namely the optimal climatic conditions for the target mixed forest distribution. The pink horizontal dash line indicates where the RIV of evergreen or deciduous broad‐leaved tree species is equal to 75%

We found a significant correlation between all tested climatic variables and the relative dominance of evergreen and deciduous broad‐leaved tree species, with opposite directions for the dominance of evergreen tree species relative to the deciduous counterpart (Figure 2). Specifically, RIV of evergreen species increased with: MAT at the lapse rate of 5.5% per °C; MinT at the lapse rate of *ca*. 10% per °C from −4.5 to 2.5°C and *ca*. 2% per °C at 2.5–10.5°C; and MAP at the lapse rate of *ca*. 10% per 100 mm between 900 and 1,600 mm and *ca*. 4% per 100 mm between 1,600 and 2,250 mm. The critical climatic conditions that resulted in equal dominance between evergreen and deciduous species were as follows: a MAT of *ca*. 14°C, a MinT of *ca*. −1°C, and a MAP of *ca*. 1,150 mm.

### 3.3 The relative role of climatic variables

**Figure 3:**
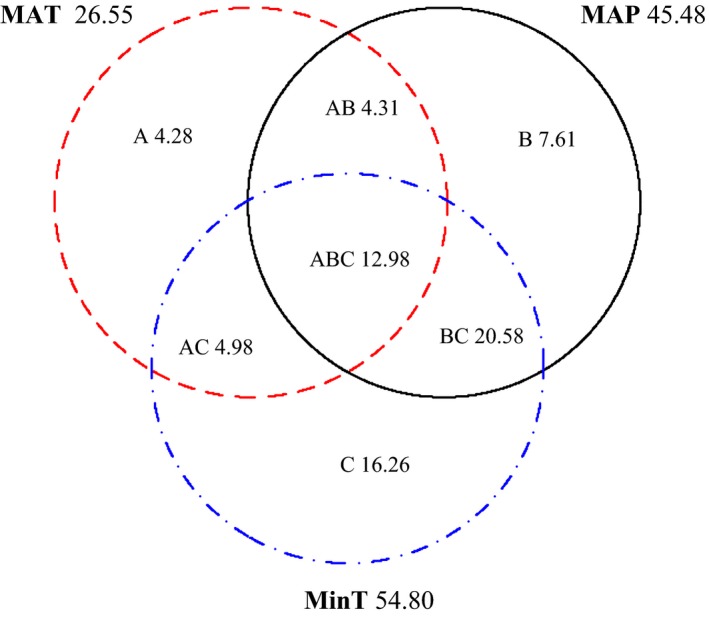
The relative contribution of different climatic variables to the variation in the relative importance value (RIV) of evergreen (or deciduous) broad‐leaved tree species in subtropical China. The symbols A, B, and C represent the independent effects of MAT, MAP, and MinT, respectively; AB is the interactive effect of MAT and MAP; AC is the interactive effect of MAT and MinT; BC is the interactive effect of MAP and MinT; and ABC is the interactive effect of MAT, MAP, and MinT. The total effect of all climatic variables is the sum of the individual and interactive effects of respective climatic variables in the corresponding circle. For example, the total effect of MAT (26.55%) is the sum of A (4.28%), AB (4.31%), AC (4.98%), and ABC (12.98%)

Variation partition analysis pointed to the extreme cold temperature (MinT) as the most influential climatic variable contributing to geographical variation in the relative dominance of evergreen versus deciduous broad‐leaved tree species (Figure 3). All tested climatic variables cumulatively explained 71% of the geographical variation. MAT independently explained only 4.28% of the total variation in evergreen versus deciduous dominance, whereas MinT was the most influential parameter, accounting for 16.26% of the variation. The interactive effect of MAP and MinT also explained a substantial proportion of the variation (20.58%). The rank of climatic variables from greatest to least total effect was as follows: MinT (54.80%) > MAP (45.48%) > MAT (26.55%).

## 4 Discussion

This study, to the best of our knowledge, is the first quantitative assessment of broad‐leaved forest community composition and its association with climatic factors in subtropical China. We found considerable overlap in the distribution of evergreen and deciduous broad‐leaved species (Figure 1). Furthermore, all tested climatic variables accounted for respectable proportions of the latitudinal variation in the dominance of species with contrasting leaf types in the broad‐leaved forests across subtropical China. These results also provide important information necessary for the definition of mixed evergreen and deciduous broad‐leaved forest.

### 4.1 Climatic correlates of evergreen versus deciduous broad‐leaved tree species distributions

We found that extreme cold temperature strongly drove directional shifts in evergreen and deciduous broad‐leaved tree species in forests across subtropical China. This result coincides well with qualitative analyses reported in earlier empirical studies that concluded the northern limit of evergreen broad‐leaved species was primarily influenced by cold temperature extremes (Box, 1995; Qian, Field, Zhang, Zhang, & Chen, 2016; Song et al., 2014). Cold tolerance and mutual competition between tree species possessing different leaf types are possible physiological mechanisms underlying this phenomenon. The biochemical processes involved in cold tolerance are energetically demanding and likely draw resources away from competitive processes (Box, 1995). Thus, an evergreen–deciduous tradeoff between cold tolerance and competitive ability exists, and greater cold tolerance can result in reduced competitive ability for either leaf type in a given climatic regime (González‐Zurdo et al., 2016; Woodward et al., 2004). Subtropical evergreen broad‐leaved tree species are able to tolerate cold temperature extremes as low as −15°C, while their deciduous counterparts can survive under temperatures below −40°C (Harrison et al., 2010). Deciduous broad‐leaved tree species adapted to cold winters in high‐latitude regions seldom thrive and even fail to reproduce in the absence of cold hardening in winter and dehardening in spring at low‐latitude areas (Harrison et al., 2010; Kreyling, Schmid, & Aas, 2015). On the other hand, evergreen broad‐leaved trees appear to possess a competitive advantage over deciduous trees at high temperatures, owing to increased carbon fixation caused by the relatively long photosynthetic periods (Lu et al., 2017; van Ommen Kloeke et al., 2012). Therefore, we assumed that minimum cold temperatures controlled northern spread of evergreen broad‐leaved species, and competition defined the southern limits of deciduous broad‐leaved tree species.

Water balance (e.g., potential evapotranspiration and precipitation) also plays a fundamental role in shaping distributions of tree species within various forests. In our study, we found potential evapotranspiration was highly correlated with the selected climatic variables (data not shown). In order to allow direct comparisons with other previous studies, we did not include this climatic variable in our analyses. Our results suggest that MAP strongly contributed to the observed distribution of evergreen and deciduous broad‐leaved tree species across our study region. This finding agreed well with earlier qualitative studies (Fang, Song, Liu, & Piao, 2001; Liu, 1997). Pronounced differences in plant water‐related traits such as water‐use strategies between leaf types likely account for this phenomenon. For example, stem and leaf hydraulic traits differ among leaf types, resulting in different water‐use strategies when evergreen and deciduous species co‐occur (Choat et al., 2011; Fan et al., 2011). Specifically, deciduous tree species display higher stem hydraulic efficiency and evergreen tree species have greater xylem‐cavitation resistance and water‐stress tolerance (Kröber, Heklau, & Bruelheide, 2015; Simonin, Limm, & Dawson, 2012). These different adaptive strategies between leaf types in response to altered precipitation patterns may scale up to influence the relative dominance of evergreen and deciduous species along the precipitation gradient within forests.

Surprisingly, we found MAT to accounted for only 4.28% of the variation in relative dominance of evergreen and deciduous broad‐leaved tree species, and the other two climatic variables to be more influential than MAT. This finding contradicts earlier studies that have shown mean annual temperature to be the primary determinant of leaf life span at both the species and community scale across various climate regimes (van Ommen Kloeke et al., 2012; Zhang et al., 2010). Scaling up from species‐level responses to emergent community‐level pattern should only be attempted with caution. The modest role of mean annual temperature in explaining variation in broad‐leaved forest physiognomy across subtropical China was perhaps due to the overriding effects of cold temperature. In fact, no straightforward relationships between MAT and broad‐leaved tree species distributions were identifiable in a previous study (Körner et al., 2016). Usually more physiologically relevant climatic features, such as growing season temperature and MinT, strongly correlate with MAT, but in this subtropical Chinese study, we did not find a strong correlation between MinT and MAT (*r* = .6). MAT did not completely reflect temperature influences on tree species distributions. Therefore, we concluded that mean annual temperature may not be an adequate descriptor of the geographical distribution of evergreen and deciduous species in subtropical broad‐leaved forests.

Climatic variables interacted in complex ways to drive shifts in the relative dominance of evergreen versus deciduous broad‐leaved tree species. Previous studies have shown that multiple simultaneously occurring climatic variables can synergistically interact (O'Brien, 2006; Stephenson, 1990). In this study, the interaction between MAP and MinT was much stronger than any other combinations of climatic variables in explaining the geographical distribution of evergreen and deciduous broad‐leaved tree species (Figure 3). Specifically, water availability and cold temperature extremes interacted to become the most important determinants of forest composition and physiognomy in subtropical China. Cold temperature extremes and total annual precipitation are often interrelated and likely impact physiological processes; therefore, they are probable mechanisms limiting the competitive ability, survival, and reproduction of tree species (Box, 1995; Harrison et al., 2010). For example, cold temperatures may impair tree performance through limitation of water uptake or by causing excessive water loss, which limit metabolism and other functions of evergreen species in dry environments (Stephenson, 1990; Zanne et al., 2014). Some studies have also shown the northern limit of evergreen broad‐leaved tree species in subtropical China to be restricted by low precipitation levels (Song, 1999). Our results suggest that mean annual precipitation can modulate the influence of MinT on relative dominance of evergreen and deciduous tree species and associated forest physiognomy. Future work that focuses on establishing the physiological processes that are responsible for the interactive effects of MAP and MinT may offer further insight into the potential mechanisms underlying the geographical distribution of tree species with different leaf types (Körner et al., 2016; Kreyling et al., 2015; Xie et al., 2015).

### 4.2 Implications for defining mixed evergreen and deciduous broad‐leaved forests

#### 4.2.1 Global definition of mixed forests

Existing definitions and quantitative indicators for different mixed forest types, including mixed evergreen and deciduous broad‐leaved forests, remain largely unclear (Bravo‐Oviedo et al., 2014; Drössler, 2010). Previous studies have tried to use a set of qualitative and quantitative vegetation characteristics, such as the number of stems, basal area, and/or biomass or canopy cover of component species, to describe and define mixed forests (Food and Agriculture Organization of the United Nations, 2002). These studies have provided some insight into development of definitions of mixed forests. However, an explicit definition for mixed forests has never been proposed, and studies have been inconsistent in choosing quantitative indicators. The first obstacle in using quantitative indicators in defining mixed forests is that there is an extensive and variable selection of proposed quantitative indicators, most of which have been developed for forest management, thus providing limited applicability to vegetation classification. For example, in an attempt to identify mixed forests, many studies have quantified wood volume and biomass of component tree species, which do not comprehensively characterize the functional roles of these component species in forest development. Moreover, some quantitative indicators, such as canopy cover, are not easily measurable (Bravo‐Oviedo et al., 2014; Jennings, Brown, & Sheil, 1999). The second problem is that there is no consensus on the cutoff levels of specific indicators in defining the mixed forests. For example, some studies have tried to differentiate mixed forests from pure forests using a threshold of 95% of volume for component species, while others have used a cutoff level 75% of volume to identify mixed forests (Bravo‐Oviedo et al., 2014; Drössler, 2010). Due to the lack of standard of a definition for mixed forests, different authors have suggested markedly inconsistent distributions of mixed forests. Therefore, unanimously agreed upon quantitative indicators and corresponding thresholds were necessary to harmonize the definition of mixed forests at global and regional scales (De Cáceres et al., 2015; Woodward et al., 2004).

A straightforward approach to define mixed forests is to utilize a quantitative indicator with a corresponding cutoff to characterize the fraction of the component species that influence forest physiognomy (Bravo‐Oviedo et al., 2014; Faber‐Langendoen et al., 2014). Here, in order to standardize quantification of the variation in component species within a mixed forest, we proposed that the RIV of tree species serves as a suitable indicator. This index has been widely adopted in the development of vegetation classification schemes because it integrates species richness, abundance, and biomass (or basal area) and provides a balanced estimate of the structural and functional role of tree species within a forest (Franklin et al., 2015; Wu, 1980).

#### 4.2.2 Definitions for mixed evergreen and deciduous broad‐leaved forest and associated climatic envelopes: Comparisons with previous studies

The mixed evergreen and deciduous broad‐leaved forest represents an important vegetation type in subtropical and warm‐temperate regions, but the recognition of this forest type remains controversial and variable among study regions and scientists (The Editorial Board of Chinese Forests, 2003). For example, in Japan this forest type has been classified into deciduous broad‐leaved forests (Box & Fujiwara, 2015), while in China this forest is considered a unique zonal vegetation type at the geographical transition zone between subtropical and temperate regions and an important forest type along the altitudinal gradients in subtropical montane regions (The Editorial Board of Chinese Forests, 2003). A major obstacle to developing a common nomenclature for this forest type arises from the lack of explicit and consistent quantitative definition for the mixed forest, despite the clear distinguishing characteristic of codominance of evergreen and deciduous broad‐leaved tree species (Wu, 1980).

Here, based on previous vegetation nomenclature rules (Wu, 1980), our proposed definition of the mixed forest, and findings from prior studies (Bravo‐Oviedo et al., 2014; Yan, 1992), we propose that the Relative Important Value index (RIV) be used as a quantitative indicator to delimit mixed forests. We propose an RIV of evergreen (or deciduous) broad‐leaved tree species that is between 25% and 75% to define the mixed evergreen and deciduous broad‐leaved forest. Additionally, in the optimum mixed forest, the proportion of evergreen broad‐leaved tree species is equal to that of their deciduous counterparts. Following the above‐mentioned reference definition of the mixed forest, we identified the latitudinal range and climatic thresholds of mixed forests in our study site. We found that the estimated latitudinal range of the mixed forests in our study site was *ca*. 29.1–32°N and the optimum distribution for this forest type occurred at *ca*. 31.39°N, where evergreen species were equal in dominance to deciduous species (Figure 1). The potential northern boundary identified here is slightly narrower than the boundary described by Zhang and Zhang (1979) (33.42–33.75°N), but it approaches the traditional boundary of Qinlin–Huaihe north–south division of China (Wu, 1980; Zhang et al., 2010). The potential southern boundary for mixed forest in our study site was much lower than the estimated boundary of the map of Chinese vegetation divisions published in Wu (1980) (*ca*. 30.3°N). These differences in boundary delimitation among studies may be ascribed to the complex topographic effects in subtropical China (Wu, 1980) and the distinct methodologies used to delimit the boundaries (Song et al., 2014; Zhang et al., 2010).

We found MAT within the optimum distribution location for the mixed forest type to be about 14°C and the corresponding temperature range to be 11–18.1°C (the lower limit was extrapolated from our regression model) (Figure 2). MinT within the optimal distribution location for the mixed forest type approached −1°C, which coincides closely with the value reported by Ohsawa (1990), while MinT range was −2.5 to 2.5°C, which is a wider temperature range than that described by Fang and Yoda (1989). The lower limit of MinT for the mixed forest type in our study site was also higher than that proposed by Box and Fujiwara (2015) for the upper limit of warm‐temperate deciduous forests in the Northern Hemisphere (−5°C). Mean annual precipitation of the optimal distribution location for the mixed forest in our study site was about 1,150 mm, and the precipitation limit was 1,000–1,630 mm, which is significantly different from the findings of Fang and Yoda (1989). The inconsistencies in findings among studies can be attributed to two possible sources of error. First, we applied a clear quantitative definition of the mixed forest, whereas Fang and Yoda (1989) delimited forest types qualitatively from a Chinese vegetation map for and applied no clear definition of the mixed forest. Second, the climatic data used in our study were derived from the world climate database, but Fang and Yoda (1989) extracted climatic data from 269 weather stations in China.

## 5 Concluding Remarks

Our analyses have assessed the geographical distribution of evergreen and deciduous broad‐leaved tree species in relation to key climatic factors. We found the extreme cold temperature to be the most important factor shaping the distribution of evergreen and deciduous broad‐leaved tree species across the Chinese subtropical region. Rainfall also played a secondary role influencing species composition within the forests across this region. Furthermore, we proposed that the RIV could serve as a quantitative indicator to define mixed forests, with a cutoff level between the mixed forest and evergreen (or deciduous) broad‐leaved forests corresponding to 25%–75% RIV.

Our work provided a synthetic approach to improve our understanding of the relative importance of and interactions among climatic factors that control the broad spatial patterns of evergreen and deciduous broad‐leaved tree species in broad‐leaved forests across subtropical China. Our study also represents a first attempt to quantitatively recognize the mixed evergreen and deciduous broad‐leaved forests as a consistent, alternative forest type.
