# Supplementary material for: Geographical and climatic gradients of evergreen versus deciduous broad‐leaved tree species in subtropical China: Implications for the definition of the mixed forest
Source: Ecol Evol. 2017 Apr 13;7(11):3636–44. doi: 10.1002/ece3.2967 (PMC5468137; doi:10.1002/ece3.2967)
Supplement: Supplementary file 1 [file ECE3-7-3636-s001.doc]

**Appendix S1. A list of major reference sources references containing data used in the analyses.**

Bi S., Peng H., Cao H. & Huang Z. (2005) Community structure and species diversity of the evergreen broadleaf forest in Nanling Dadingshan Nature Reserve. *Ecological Science* **24**,113-116.

Chen L., Chen Q. & Liu W. (1997) Diversity and the geographical distribution of forests in China. Science Press, Beijing.

Chen R. &Xiao W. (2000) A study on *Quercus Serrata* community characteristics in Baotianman area, Henan Province. *Scientia Silvae Sinicae* **36**:21-25.

Ge J., Hu H. & Li B. (2009). Scientific Survey and Study on Forest Biodiversity in Mulinzi Nature Reserve of Hubei, Central China. Science Press, Beijing.

Ge J., Xiong G., Deng L., Zhao C., Shen G. & Xie Z. (2012) Community dynamics of a montane *Fagus engleriana–Cyclobalanopsis multinervis* mixed forest in Shennongjia, Hubei, China. *Biodiversity Science* **20**, 643-653.

Ge, J., Xiong, G., Wang, Z., Zhang, M., Zhao, C., Shen, G., Xu, W. & Xie, Z. (2015) Altered dynamics of broad-leaved tree species in a Chinese subtropical montane mixed forest: the role of an anomalous extreme 2008 ice storm episode. *Ecology and Evolution* **5**, 1484-1493.

He H. & He X. 2003. A numerical and synthetic study of evergreen and broad-leaved tree species in Shunhuang Mountain of Hunan Province: I. study of species significant value. *Journal of Central South Forestry University* **23**, 16-21.

He H. & He X.2005. A synthetic study of evergreen and broadleaf trees in Shunhuang Mountain of Hunan Province: Ⅳ.Study of Species Diversity. *Journal of Central South Forestry University* **25**, 42-46.

Huang B. (2006) Structural characteristics of the evergreen broadleaved forest as *Castanopsis carlesii* is establishing species in Erdu, Fujian. *Journal of Fujian Forestry Science and Technology* **33**, 6-10

Huang C. (1986) Studies on the population structure and succession trend of evergreen coniferous-broadleaf forests on the north slope of Jiugong Mountain. *Chinese Journal of Ecology* **5**, 31-36.

Huang Y., Yao L., Ai X., Lv S. & Ding Y. (2015) Quantitative classification of the subtropical evergreen-deciduous broadleaved mixed forest and the deciduous and evergreen species composition structure across two national nature reserves in the southwest of Hubei, China. *Chinese Journal of Plant Ecology* **39**, 990–1002.

Jian M. & Liu Q. (2008) Structural characteristics of evergreen broad-leaved forest in Jiulianshan Mountain of Subtropical China. *Jiangxi Science* **26**,198-211.

Jiang G. & Wu Z. (2011) *Castanopsis eyrei* community characteristics in Zhawan Natural Reserve of Anhui Province. *Journal of Anhui Agricultural University* **38**, 517-521.

Jiang Y., Guo Q. & Ma J. (1998) Classification and characteristics of Chinese forests. Science Press and China Forestry Publishing House, Beijing.

Kang M., Xie Y., Xu Y., Xu Y. & Yan E. (2013) Within-community variability of plant leaf N and P contents in Tiantong, Zhejiang Province. *Journal of East China Normal University* (*Natural Science*) **2**, 20-29.

Kong B., Cao H., Ma L., Wu L., Chen C., Huang Z. (2013) Community Characteristics of the Fengshui-wood of *Erythrophleum Fordii* in Guangzhou. *Tropical Geography* **33**, 307-313.

Li J., Li G., Yue Z. & Li R. (1990) Evergreen components in an evergreen and deciduous broad-leaved mixed forest in Maoping area: implication for management and utilization. *Shaanxi Forest Science and Technology* **3**, 22-24.

Li L. (2005) The Community Characteristic of Evergreen Broad-leaved Forest in Linnan Bandong Nature Reserve, Guangdong Province. *Guangdong Forestry Science and Technology* 21:39-43.

Li X., Tian Y., Hu L., Huang H. & Jiang M. (2002) Community Characteristic of the Mixed Eevergreen and Deciduous Broadleaved Forest in Houhe Nature Reserve. *Journal of Wuhan Botanical Research* **20**, 353-358.

Liu J. (1989) The application of Fuzzy Cluster method to the classification of plant community. *Journal of Shaanxi Normal University* (*Natural Science Edition*) **17**, 57-62.

Liu J. (1991) Ordination of the evergreen broad-leaf forest of Beiba in Shaanxi. *Journal of Shaanxi Normal University* (*Natural Science Edition*) 19, 64-68

Liu Y., Yue Q. & Huang L. (1993) The properties of plant community of the fixed plot of the evergreen broad-leaved forest in Sanctuary of Jinyun Mountain. *Journal of Southwest China Teachers University* (*Natural Science*) **18**, 76-84.

Lu Z., Bao D., Guo Y., Lu J., Wang Q., He D., Zhang K., Xu Y., Liu H., Meng H., Huang H., Wei
X., Liao J., Qiao X., Jiang M., Gu. Z. & Liao C. (2013) Community composition and structure of Badagongshan (BDGS) forest dynamic plot in a Mid-subtropical Mountain Evergreen and Deciduous Broad-leaved Mixed Forest, Central China. *Plant Science Journal* **31**, 336-344.

Luo G. & Ye Y. (1990) Community characteristics of Evergreen broad-leaved forests in Daiyunshan natural Reserve. *Subtropical Plant Research Communications* **2**, 27-34.

Mu C. (2008) Studies on Vegetation and plant communities of Daguping Forestland in Hunnan Province. MS dissertation. Central South University of Forestry and Technology.

Peng D., Yuan Z., Tang C., Peng G.. & Liao Q. (1985) Characteristic analyses of evergreen broadleaf trees in Dongtinghu area. *Chinese journal of Ecology* **3**, 16-21.

Qi C. (2011) Comprehensive studies on Subtropical vegetation of China. China Forestry Publishing House, Beijing.

Shen Z., Hu H., Zhou Y. & Fang J. (2004) Altitudinal patterns of plant species diversity on the southern slope of Mt. Shennongjia, Hubei, China. *Biodiversity Science* **12**, 99-107.

Song K. (2012) Vegetation Characteristic of evergreen-deciduous broadleaved forest ecotone and its formation mechanism, a study in Anhui. PhD dissertation. East China Normal University.

Song Y. (2013) Evergreen broad-leaved forests in China: Classification, Ecology and Conservation. Science Press, Beijing.

Song Y., Zhang S., Wang X., Liu J., Gu Y. & Hu S. (1982) Community analysis of the evergreen broad-leaved forest on Mountain Wuyanling in Zhejiang Province. *Acta Phytoecologica et Geobotanica Sinica* **6**, 14-35.

Song, K., Kohyama, T.S. & Da, L.J. (2014) Transition patterns across an evergreen–deciduous broad-leaved forest ecotone: the effect of topographies. *Journal of Vegetation Science* 25, 1257-1266.

Tang C. (2015) The Subtropical Vegetation of Southwestern China: Plant Distribution, Diversity and Ecology. Springer.

The Editorial Board of Chinese Forests (2003) Chinese Forests (Volume 3: Broad-leaved Forests). China Forestry Publishing House, Beijing.

Wang Q. (2012) Study on community characteristics of *Phoebe zhennan* in Enshi, Hubei Province. MS dissertation, Zhejiang A&F University.

Wang Y. (2011) Dynamics of community structure and regeneration characteristics of major populations in an evergreen broad-leaved forest in Gutianshan National Natural Reserve, east China. MS dissertation. Zhejiang University.

Xie Z. & Chen W. (1998) The remaining evergreen broad-leaved forest and its significance in the Three Gorges Reservoir Area. *Acta Phytoecologica Sinica* 22,422-427.

Xu M.(2006) Studies on community characters of the mid-montane evergreen broad-leaved forest in Baishanzu Mountain. MS dissertation. Zhejiang University.

Yan C. (1992) Study on the characteristics of evergreen and deciduous broad-leaved mixed forest. *Journal of Xuzhou Teachers College* **10**, 27-31.

Yang X., Xing F., Chen S. & Zeng Q. (2013) Structure Characteristics of *Manglietia pachyphylla* Community in Nankunshan Nature Reserve, Guangdong Province. *Journal of Tropical and Subtropical Botany* **21**, 356-364.

Yang Y. & Li T. (1989) Preliminary studies on the phytocommunity of *Davidia involucratain* Omei Mountain of Sichuan. *Acta Phytoecologica et Geobotanica Sinica* 13, 270-276.

Yang Y. (2013) Species composition and distribution pattern of the typical evergreen broad-leaved forest in Wuyi Mountains. MS dissertation. Nanjing University of Information Science and Technology.

Yang Y., Zhuang P. & Li X. (1994) Ecological studies on the forest community of *Castanopsis platyacantha -Schima sinensis* on Emei Mountain. *Acta Phytoecologica Sinica* **18**,105-120.

Zhang S. & Zheng S. (2001) Structural features of evergreen broad-leaved forest community in Bijia Mountain of Longyan,Fujian. *Scientia Silvae Sinicae* 37, 111-116.

Zhang Y., Wu L. & Zhao T. 1996. Study on the major types of vegetation and their vertical distribution in Baotianman Natural Reserve in Henan. *Acta Agriculturae Universitatis Henanensis* 30:178-185.

Zhao L. (2014) Structure characteristics and influencing factors of *Lithocarpus glaber*-*Cyclobalanopsis glauca* community in subtropical evergreen broad-leaved forest. PhD dissertation, Central South University of Forestry and Technology.

Zhao L., Xiang W., Li J., Deng X. & Liu C. (2013) Floristic composition, structure and phytogeographic characteristics in a *Lithocarpus glaber*-*Cyclobalanopsis glauca* forest community in the Subtropical Region. *Scientia Silvae Sinicae* **49**, 10-17.

Zheng S. 2013. Studies on plant diversity and its evaluation of Fujian Daiyun Mountain National Nature Reserve. PhD dissertation. Fujian Agriculture and Forestry University.

Zhou S., Liu Y., Jiang J. (1996) A preliminary study on two main types of vegetation from Zawan, Qimen County, Anhui Province. *Journal of Xuzhou Teachers College* **14**, 43-48.

Zhou S., Peng Y., Huang H. & Jiang M. (2010) Dynamics of major tree species in a rare plant community over 6 years in Houhe Nature Reserve, Central China. *Journal of Wuhan Botanical Research* **28**, 315-323.

Zhu Y., Zhao G., Zhang L., Shen G., Mi X., Ren H., Yu M., Chen J., Chen S., Fang T. & Ma K. (2008) Community composition and structure of Gutianshan forest dynamic plot in a mid-subtropical evergreen broad-leaved forest, east China. *Chinese Journal of Plant Ecology* **32**, 262-273.

Zuo J., Tian W., Peng D., Li M. & Peng Z. (2001) Original Forest Community in Nanyue of Hunan ProvinceⅠ. *Castanopsis eyeri* Forest. *Journal of Hunan Agricultural University* (*Natural Sciences*) **27**, 121-126.

Zuo J., Zhu X., Tian W., Peng D. & Li M. (2001) Study on Original Forest Community in Nanyue of Hunan Province Ⅲ. Lithocarpus henryi Forest. Journal of Wuhan Botanical Research 19, 377-38.

Zuo J., Zhu X., Peng D. & Li M. (2004) Study on original forest community in Nanyue of Hunan Province-Ⅳ. *Lithocarpus cleistocarpus* and *Quercus aliena* var*. acuteserrata* forest. *Guihaia* **24**, 224-231.

**Appendix S2. Additional information on the study region and mixed evergreen and deciduous broad-leaved forests.**

**1. Additional information on the study region and mixed evergreen and deciduous broad-leaved forests**

The study region was located in subtropical China and ranged from 23.5°to 34.0°N in latitude and from 103.1° -121.8° E in longitude (**Figure S1**). According to the climate classification system, the subtropical zone is adjacent to the (warm-) temperate zone. However, the geographical ranges of the subtropical and temperate zones are considerably different and may overlap, depending on the climate zone classification systems applied. Some controversy has arisen in delineation of the boundary between subtropical and warm-temperate regions. In China, the subtropical zone generally ranges from 22-34°N (The Editoral Commette of Vegetation Map of China, 2007), but other climate classification systems, e.g. such as Holdridge (1947), assign a more narrow range to this zone. One striking difference between Chinese climate classification systems and other classification systems is that Chinese classifications systems assign thermal zonation based on the number of days of the year with average daily temperature ≥10℃; other classification systems are based on climate conditions through the entire year. This≥10℃index for daily temperature represents the temperature threshold for plant sprouting; it is an important climate variable used in forestry and agriculture management and is widely adopted by Chinese meteorologists and vegetation scientists (Zhu, 1984). Recent terrestrial ecology research on the geographic range of the subtropics has indicated that the agreed international definition of the subtropics is about 23.4-30.0° (Corlett, 2013).

**Figure S1** Geographical distribution of broad-leaved forest plots in this study

**2. Mixed evergreen and deciduous broad-leaved forests and the list of dominant tree species within the studied region**

Distributions of evergreen and deciduous broad-leaved tree species can overlap and form various vegetation climaxes (evergreen forests, mixed forests, and deciduous forests) in some regions (Woodward et al 2004; Wu 1980). The typical mixed forest is considered to be composed of transitional vegetation between evergreen and deciduous forests; therefore, shifts in species abundance should be typical and distinct in this mixed forest. The typical mixed forest also connects evergreen and deciduous broadleaved forests in some regions (Ge et al 2016). Climate change is anticipated to shift the ranges of evergreen and deciduous broad-leaved tree species, resulting in expansion and/or contraction of various broad-leaved forests. Therefore, mixed forests are extremely vulnerable to climate fluctuations (Wu 1980). However, the boundaries of these forests have not been well defined (Song et al 1999; The Editorial Board of Chinese Forests 2003). The typical mixed forest is located at the transitional zone between the subtropical and warm-temperate zone and spans a much wider latitudinal range in China than in other regions in the world (Liu 1997). To some degree, the mixed forest is peculiar to and typical of subtropical China. Nevertheless, a quantitative ecological definition of this forest type does not exist, and thus misconceptions are common when this type of forest is studied among different vegetation ecologists. Therefore, quantification of the typical mixed forest is of key importance in studying the climate-vegetation relationship.

We have listed the dominant tree species in this study below. And a more detailed list of tree species found in typical mixed evergreen and deciduous broad-leaved forests can be found in Ge et al (2013).

*Acer oblongum, Bothrocaryum controversum, Carpinus turczaninowii, Carpinus viminea, Castanopsis eyrei, Castanopsis hystrix, Castanopsis sclerophylla* , *Castanopsis platyacantha, Cyclobalanopsis chungii, Cyclobalanopsis fargesii, Cyclobalanopsis glauca, Cyclobalanopsis multinervis, Cyclobalanopsis myrsinaefolia, Cyclobalanopsis sessilifolia, Cyclobanopsis oxyodon, Daphniphyllum macropodum, Fagus engleriana, Fagus longipetiolata, Fagus lucidum, Fagus pashanica, Ilex chinensis, Liquidambar acalycina, Lithocarpus cleistocarpus, Quercus aliena* var*．acuteserrata, Quercus engleriana, Quercus glandulifera, Quercus serrata, Quercus variabilis, Sassafras tsumu, Schima superba,* and *Toxicodendron vernicifluum.*

**3. References containing additional information on the studied region**

Box EO, Fujiwara K (2015) Warm-temperate deciduous forests around the Northern Hemisphere. Springer.

Corlett RT (2013) Where are the Subtropics? Biotropica 45:273-275.

Ge J, Xie Z, Xu W, Zhao C (2016) Controls over leaf litter decomposition in a mixed evergreen and deciduous broad-leaved forest, Central China. Plant Soil:1-11 doi:10.1007/s11104-016-3077-9.

Ge J, Xiong G, Zhao C, Shen G, Xie Z (2013) Short-term dynamic shifts in woody plants in a montane mixed evergreen and deciduous broadleaved forest in central China. Forest Ecol Manag 310:740-746.

Holdridge LR (1947) Determination of world plant formations from simple climatic data. Science 105:367-368.

Liu C (1997) The distribution of evergreen broad-leaved forest in East Asia. Nat Res 19: 36-45.

Song, Y. (1999) Perspective of the vegetation zonation of forest region in eastern China. Journal of Integrative Plant Biology 41: 541-552.

The Editoral Commette of Vegetation Map of China (2007) Geographical patterns of Chinese vegetations. Geological Publishing House, Beijing.

The Editorial Board of Chinese Forests (2003) Chinese Forests (Volume 3: Broad-leaved Forests). China Forestry Publishing House, Beijing

Woodward F, Lomas M, Kelly C (2004) Global climate and the distribution of plant biomes. Philos Trans R Soc Lond B Biol Sci 359: 1465-1476.

Wu, Z. (1980) Vegetation of China. In. Science Press, Beijing.

Zhu K (1984) Physical Geography of China. Science Press, Beijing.

**Appendix S3. Selection of climatic variables and associated references.**

**1. Selection of climatic variables**

Climatic data for each site was acquired from WorldClim (http://www.worldclimate.org/) (Hijmans et al., 2005). Given the strong main effects and potential for interactions and colinearity among climatic variables, we limited our climatic analyses to: mean annual temperature, minimum temperature of the coldest month, and mean annual precipitation. These variables were chosen based a principal component analysis (PCA) (**See Table S1**) and previous broad-scale studies, which found these climatic variables to influence plant attributes and the distribution of evergreen and deciduous tree species (Skai, 1979; Ohsawa, 1990; O'Brien, 2006; Harrison et al., 2010; Zhang et al., 2010; Kikuzawa and Lechowicz, 2011; van Ommen Kloeke et al., 2012; Kikuzawa et al., 2013; Moles et al., 2014; Song et al., 2014; Shiono et al., 2015). Furthermore, potential evapotranspiration is usually modeled using the data available from WorldClim Global Climate Data (Zomer et al., 2008) (http://www.cgiar-csi.org/data/global-aridity-and-pet-database). Thus, potential evapotranspiration for our study sites was highly correlated with MAP. So we did not incorporate this climatic variable into our analysis.

**2. References cited in the ‘Selection of climatic variables’ section**

Harrison SP, Prentice IC, Barboni D, Kohfeld KE, Ni J, Sutra J-P (2010) Ecophysiological and bioclimatic foundations for a global plant functional classification. J Veg Sci 21:300-317 doi:10.1111/j.1654-1103.2009.01144.x.

Hijmans RJ, Cameron SE, Parra JL, Jones PG, Jarvis A (2005) Very high resolution interpolated climate surfaces for global land areas. Int J Climatol 25:1965-1978.

Kikuzawa K, Lechowicz MJ (2011) Ecology of leaf longevity. Springer Science & Business Media

Kikuzawa K, Onoda Y, Wright IJ, Reich PB (2013) Mechanisms underlying global temperature-related patterns in leaf longevity. Global Ecol Biogeogr 22:982-993.

Moles AT et al. (2014) Which is a better predictor of plant traits: temperature or precipitation? J Veg Sci 25:1167-1180 doi:10.1111/jvs.12190.

O'Brien EM (2006) Biological relativity to water–energy dynamics. J Biogeogr 33:1868-1888.

Ohsawa M (1990) An interpretation of latitudinal patterns of forest limits in south and east Asian mountains. J Ecol:326-339.

Shiono T, Kusumoto B, Maeshiro R, Fujii S-J, Götzenberger L, de Bello F, Kubota Y (2015) Climatic drivers of trait assembly in woody plants in Japan. J Biogeogr 42:1176-1186.

Skai A (1979) Freezing tolerance of evergreen and deciduous broad-leaved trees in Japan with reference to tree regions. Low temperature science Ser B, Biological sciences 36:1-19.

Song K, Kohyama TS, Da LJ (2014) Transition patterns across an evergreen–deciduous broad-leaved forest ecotone: the effect of topographies. J Veg Sci 25:1257-1266 doi:10.1111/jvs.12156.

van Ommen Kloeke A, Douma J, Ordoñez J, Reich P, Van Bodegom P (2012) Global quantification of contrasting leaf life span strategies for deciduous and evergreen species in response to environmental conditions. Global Ecol Biogeogr 21:224-235.

Zhang L, Luo T, Zhu H, Daly C, Deng K (2010) Leaf life span as a simple predictor of evergreen forest zonation in China. J Biogeogr 37:27-36.

Zomer, R.J., Trabucco, A., Bossio, D.A. & Verchot, L.V. (2008) Climate change mitigation: A spatial analysis of global land suitability for clean development mechanism afforestation and reforestation. Agr Ecosyst Environ 126: 67-80.

| **Table S1** Principal component analysis results for climatic variables across all sampling locations in this study | | | |
| --- | --- | --- | --- |
|  | **Component1** | **Component2** | **Component3** |
| **Standard deviation** | 2.77 | 2.05 | 1.79 |
| **Proportion of variance** | 0.40 | 0.22 | 0.17 |
| **Cumulative proportion** | 0.40 | 0.62 | 0.79 |
| **Loadings** |  |  |  |
| Mean annual temperature | 0.28 | 0.25 | -0.10 |
| Mean monthly temperature range | -0.10 | 0.18 | -0.14 |
| Isothermality | 0.12 | -0.16 | -0.40 |
| Temperature seasonality | -0.20 | 0.30 | 0.31 |
| Max temperature of the warmest Month | 0.15 | 0.42 | 0.12 |
| Min Temperature of the coldest month | 0.32 | 0.05 | -0.17 |
| Temperature Annual Range | -0.19 | 0.31 | 0.28 |
| Mean Temperature of Wettest Quarter | -0.04 | 0.23 | 0.24 |
| Mean Temperature of driest Quarter | 0.32 | 0.11 | -0.15 |
| Mean Temperature of warmest Quarter | 0.15 | 0.42 | 0.10 |
| Mean Temperature of coldest Quarter | 0.32 | 0.07 | -0.21 |
| Mean annual precipitation | 0.29 | -0.17 | 0.23 |
| Precipitation of wettest month | 0.04 | -0.30 | 0.33 |
| Precipitation of driest month | 0.27 | -0.01 | 0.18 |
| Precipitation seasonality | -0.28 | -0.04 | -0.05 |
| Precipitation of wettest Quarter | 0.18 | -0.26 | 0.31 |
| Precipitation of driest Quarter | 0.28 | 0.04 | 0.25 |
| Precipitation of warmest Quarter | 0.08 | -0.27 | 0.31 |
| Precipitation of coldest Quarter | 0.32 | 0.00 | 0.09 |
| **Eigenvalue** | 7.67 | 4.19 | 3.21 |

**Appendix S4. Additional information on Data analyses and Processing.**

**1. Supplementary materials for Data analyses and Processing**

In our statistical analysis, we only presented the results of the relationships between the Relative Importance Value (RIV) of evergreen broad-leaved tree species and the selected geographical and climatic variables. The statistical models constructed for deciduous broad-leaved tree species showed the same patterns as the models for deciduous species, because the RIV of evergreen and deciduous broad-leaved tree species summed to 100%.

To examine latitudinal and climatic patterns between EBF and DBF across the subtropical region, we conducted multiple regression analysis by using the RIV of EBT and DBT as response variables, and latitude and MinT, MAT, MAP, and their respective square and cubic functions as explanatory variables. Before fitting the model, we identified the potential independent variables through review of previous studies (Lusk et al. 2016; Messaoud et al. 2007; Paruelo and Lauenroth 1996; Song et al. 2014; Suzuki et al. 2015; Zhang et al. 2010). For example, Suzuki et al (2015) showed that the relative abundance of evergreen and deciduous tree species in Japanese broad-leaved forests can be precisely modeled using MAT and MAT^2^ (square of MAT). Song (2014) reported a similar relationship across an evergreen-deciduous broad-leaved forest ecotone. We thus incorporated MAT and MAT^2^ into our statistical analysis. Consequently, the relationships between the response and explanatory variables were fitted using linear, second-order polynomial, and third-order polynomial models. The inclusion of quadratic and cubic terms allowed for modeling nonlinear responses. Only those variables that significantly improved the fit of the model were included. We selected the best-fit models by calculating Akaike’s Information Criterion (AIC) and following the methods of previous similar studies (Song et al. 2014; Suzuki et al. 2015). The model with the lowest AIC values among all models within the candidate set of models was considered to possess the greatest support (Aho et al. 2014; Murphy and Bowman 2007). The detailed statistical results are shown in Table S2. As shown in this table, we have selected the third-order polynomial models to explore the relationship between the RIV of evergreen broad-leaved tree species and latitudinal and climatic variables. For MAT, Analysis of Variance (p>0.05) showed no significant difference among the three models, so we selected the third-order polynomial model to maintain consistency with the other climatic variables, such as MinT. In this study, we adopted third-order polynomial models to explore the relationships between the relative distribution of evergreen and deciduous broad-leaved tree species and latitude and climatic variables.

Next, we conducted a variation partitioning analysis to parse the relative contribution of various climatic variables to the variation in the relative distribution of evergreen and deciduous broad-leaved tree species. Before this analysis, we used forward stepwise model selection methods to construct the best-fit models using the RIV of evergreen broad-leaved tree species as response variables and the above-mentioned climatic variables as potential independent variables (Nathans et al. 2012). Then the variation in the RIV of evergreen broad-leaved tree species was partitioned into pure and interactive effects of the above-mentioned climatic variables. We applied variation partitioning analysis following the statistical techniques provided by Ray-Mukherjee et al. (2014). We also performed variation partitioning analyses using other statistical models, which excluded the quadratic and cubic terms of climatic variables, in order to further disentangle the relative importance of climatic variables. All tested models produced similar results and thus verified our current conclusion.

| **Table S2** Statistical results from our fitted models between the RIV of evergreen broad-leaved tree species and geographical and climatic variables | | | |
| --- | --- | --- | --- |
| **Variables included** | **R^2^** | **p value** | **AIC** |
| **RIV of the evergreen versus Latitude** |  |  |  |
| Latitude | 0.5208 | <0.01 | 447.3681 |
| Latitude, Latitude^2^ | 0.7130 | <0.01 | 447.3681 |
| Latitude, Latitude^2^, Latitude^3^ | 0.7572 | <0.01 | 440.3349 |
| **RIV of the evergreen versus MAT** |  |  |  |
| MAT | 0.2630 | <0.01 | 497.3052 |
| MAT, MAT^2^ | 0.2764 | <0.01 | 499.2397 |
| MAT, MAT^2^, MAT^3^ | 0.2773 | <0.01 | 499.2397 |
| **RIV of the evergreen versus MAP** |  |  |  |
| MAP | 0.4548 | <0.01 | 462.3107 |
| MAP，MAP^2^ | 0.6215 | <0.01 | 462.3107 |
| MAP，MAP^2^，MAP^3^ | 0.6720 | <0.01 | 456.5803 |
| **RIV of the evergreen versus MinT** |  |  |  |
| MinT | 0.5480 | <0.01 | 469.8959 |
| MinT, MinT^2^ | 0.6679 | <0.01 | 455.2569 |
| MinT, MinT^2^, MinT^3^ | 0.6750 | <0.01 | 456.0899 |

**2. References cited in supplementary sections**

Aho K, Derryberry D, Peterson T (2014) Model selection for ecologists: the worldviews of AIC and BIC. Ecology 95:631-636.

Lusk CH, McGlone MS, Overton JM (2016) Climate predicts the proportion of divaricate plant species in New Zealand arborescent assemblages. J Biogeogr 43:1881-1892.

Messaoud Y, Bergeron Y, Leduc A (2007) Ecological factors explaining the location of the boundary between the mixedwood and coniferous bioclimatic zones in the boreal biome of eastern North America. Global Ecol Biogeogr 16:90-102.

Murphy BP, Bowman DM (2007) Seasonal water availability predicts the relative abundance of C3 and C4 grasses in Australia. Global Ecol Biogeogr 16:160-169

Nathans LL, Oswald FL, Nimon K (2012) Interpreting multiple linear regression: A guidebook of variable importance. Pract Asse, Res Eval 17: 2.

Paruelo JM, Lauenroth W (1996) Relative abundance of plant functional types in grasslands and shrublands of North America. Ecol Appl:1212-1224

Ray-Mukherjee J, Nimon K, Mukherjee S, Morris DW, Slotow R, Hamer M (2014) Using commonality analysis in multiple regressions: a tool to decompose regression effects in the face of multicollinearity. Meth Ecol Evol 5:320-328.

Song K, Kohyama TS, Da LJ (2014) Transition patterns across an evergreen–deciduous broad-leaved forest ecotone: the effect of topographies. J Veg Sci 25:1257-1266.

Suzuki SN, Ishihara MI, Hidaka A (2015) Regional-scale directional changes in abundance of tree species along a temperature gradient in Japan. Global Change Biol 21:3436-3444.

Zhang L, Luo T, Zhu H, Daly C, Deng K (2010) Leaf life span as a simple predictor of evergreen forest zonation in China. J Biogeogr 37:27-36.
